# Cancer-associated fibroblasts as architects of acquired immune-privileged-like niches in gastrointestinal cancers: lessons from ocular immune privilege and barrier biology

**DOI:** 10.3389/fimmu.2026.1946857

**Published:** 2026-08-25

**Authors:** Yingjian Wang, Zhe Wang, Fangdi Zhou, Yu Qiao, Zhanyang Luo, Feng Wang, Bingtong Yue, Dao Xin

**Affiliations:** 1 Department of Urology, The First Affiliated Hospital of Zhengzhou University, Zhengzhou, Henan, China; 2 Department of Oncology, The First Affiliated Hospital of Zhengzhou University, Zhengzhou, China; 3 Department of Ophthalmology, The First Affiliated Hospital of Zhengzhou University, Zhengzhou, Henan, China; 4 Department of Orthopaedics and Sports Orthopaedics, TUM University Hospital, Technical University of Munich, Munich, Germany; 5 Department of Pharmacy, Shanghai Pudong Hospital, Fudan University Pudong Medical Center, Shanghai, China; 6 Department of Colorectal Surgery and Oncology, Key Laboratory of Cancer Prevention and Intervention, Ministry of Education, The Second Affiliated Hospital, Zhejiang University School of Medicine, Hangzhou, Zhejiang, China

**Keywords:** cancer-associated fibroblasts, extracellular matrix, gastrointestinal cancer, immune exclusion, immune privilege, ocular immune privilege, therapy resistance, tumor microenvironment

## Abstract

Gastrointestinal cancers frequently develop immune exclusion and therapeutic resistance, yet these phenotypes are often described separately. Cancer-associated fibroblasts (CAFs) are key stromal architects of immune-excluded tumor microenvironments, and their heterogeneous states can evolve under chemotherapy, radiotherapy, immunotherapy, targeted therapy, and anti-angiogenic pressure. We propose that treatment-induced injury, inflammation, hypoxia, and repair signals reshape CAFs. These therapy-educated CAFs can construct acquired immune-privileged-like niches by integrating extracellular matrix remodeling, immunosuppressive secretomes, vascular-barrier remodeling, and spatial exclusion of cytotoxic immune cells. Ocular barrier biology offers a conceptual, not anatomical, analog for this spatial organization. In gastrointestinal tumors, these principles may be pathologically co-opted by myCAF-, iCAF-, and apCAF-like programs to restrict CD8+ T-cell and NK-cell access, recruit suppressive myeloid and regulatory populations, and impair drug delivery. This framework may guide spatial multiomics, digital pathology, radiomics, CAF-derived biomarkers, and spatially guided CAF reprogramming strategies. It also provides falsifiable questions for paired pre- and post-treatment sampling across gastrointestinal cancer types.

## Introduction

Gastrointestinal cancers, including esophageal squamous cell carcinoma, gastric cancer, colorectal cancer, and pancreatic cancer, remain major causes of cancer mortality. Immunotherapy, chemotherapy, radiotherapy, targeted therapy, and anti-angiogenic therapy have improved outcomes in selected patients, but primary and acquired resistance remain common. One recurring phenotype is immune exclusion, in which cytotoxic lymphocytes are detectable in the tumor microenvironment but remain spatially restricted outside tumor nests. This pattern is clinically important because checkpoint blockade requires not only antigenicity and T-cell priming, but also physical access and preserved cytotoxic function within tumor tissue (1–4).

CAFs are not passive stromal bystanders. They are dynamic, heterogeneous, and therapy-responsive stromal populations that influence extracellular matrix (ECM) architecture, cytokine gradients, vascular behavior, and immune cell positioning (5, 6). Single-cell and functional studies have clarified myofibroblastic, inflammatory, and antigen-presenting-like CAF states, yet a conceptual gap remains. The field still needs a concise framework that connects stromal barrier formation, local immune restraint, vascular remodeling, drug delivery failure, and therapy-induced evolution into one testable model.

**Figure 1:**
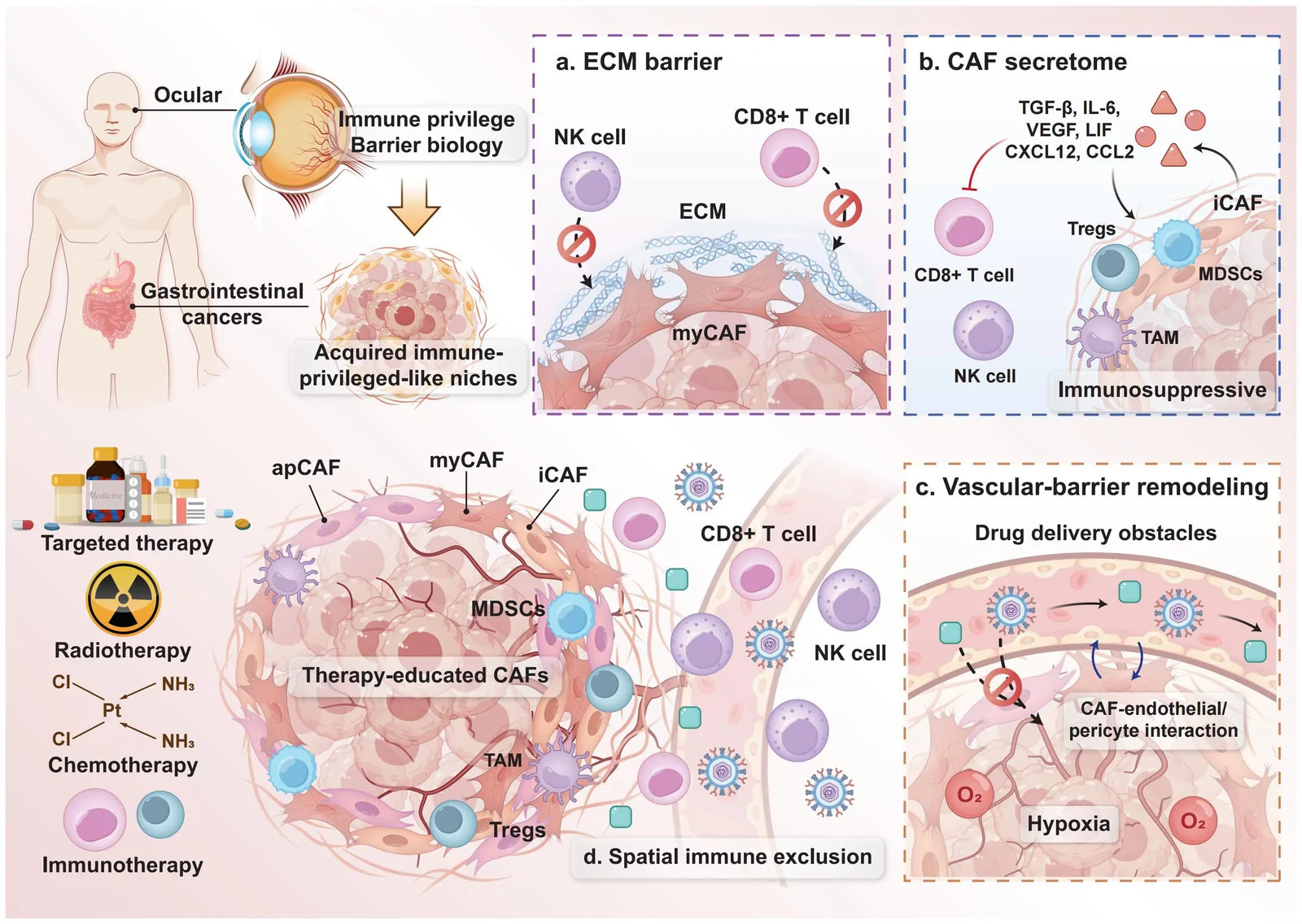
CAF-driven acquired immune-privileged-like niches in gastrointestinal cancers: lessons from ocular barrier biology. The left panel illustrates the conceptual transition from ocular immune privilege to acquired immune-privileged-like niches in gastrointestinal cancers. CAFs reprogrammed under therapeutic pressure are depicted as the central organizers of these pathological niches. **(a)** myCAFs establish an ECM barrier that restricts immune cell infiltration. **(b)** iCAFs create an immunosuppressive secretome through the release of cytokines and chemokines, promoting the accumulation of Tregs, MDSCs, and TAMs. **(c)** CAF-mediated vascular-barrier remodeling impairs endothelial function and vessel patency, limiting drug delivery and promoting hypoxia. **(d)** The coordinated actions of ECM remodeling, immunosuppressive signaling, and vascular dysfunction ultimately lead to spatial immune exclusion and therapy resistance.

We propose that CAF-rich gastrointestinal tumors can be reframed as acquired immune-privileged-like ecosystems shaped by therapy-induced stromal evolution. The analogy is deliberately bounded. Gastrointestinal tumors are not immune-privileged organs, and ocular immune privilege should not be treated as a direct anatomical or disease model. Instead, ocular barrier biology offers a conceptual lens for asking how barrier architecture, stromal remodeling, vascular regulation, and local immune restraint can generate spatially protected niches (7–10). This lens is useful because it turns a descriptive problem into a spatial and functional hypothesis: if CAFs build acquired immune-privileged-like niches, then CAF state changes should map to immune exclusion, altered vessel access, and residual disease after therapy. Here, we propose an interdisciplinary perspective that reframes CAF-rich gastrointestinal tumors as acquired immune-privileged-like ecosystems, in which stromal barriers, immunosuppressive secretomes, and vascular remodeling converge to promote immune evasion and therapeutic resistance. This conceptual framework is summarized in **Figure 1**.

## CAF heterogeneity and therapy-driven plasticity in gastrointestinal cancers

CAF heterogeneity is central to this perspective. myCAFs are commonly associated with ACTA2, TAGLN, COL1A1, COL3A1, ECM deposition, contractility, tissue stiffness, and mechanical immune exclusion. iCAFs are enriched for inflammatory and immunoregulatory mediators, including IL6, CXCL12, LIF, CXCL1/2, and related cytokine or chemokine programs. apCAFs show MHC-II-related features and may participate in antigen-presentation-like or immunoregulatory interactions. These labels are useful, but they should be interpreted as plastic state programs rather than fixed lineages (5, 11–13).

The gastrointestinal context makes this plasticity particularly relevant. Pancreatic cancer is the desmoplastic prototype, with dense matrix, CAF-rich regions, abnormal perfusion, and restricted drug penetration. Colorectal cancer has shown strong links between stromal programs, TGF-beta signaling, immune exclusion, and poor prognosis (2–4, 14–16). Gastric cancer and esophageal squamous cell carcinoma are clinically relevant settings in which stromal and immune contexture may shape treatment response and immune-checkpoint resistance, although the precise CAF programs and spatial rules will need cancer-specific validation rather than direct extrapolation from pancreatic or colorectal models. In gastric cancer, recent molecular classification studies have also linked immune-related gene signatures with tumor immune subtypes, prognosis, and microenvironmental remodeling, supporting the need to interpret CAF programs within broader immune-ecological contexts (17). In ESCC, checkpoint resistance reflects interacting tumor-intrinsic and microenvironmental mechanisms, including impaired antigen presentation, suppressive immune cells, and inhibitory cytokine networks (18). The CAF framework adds a spatial and stromal dimension without replacing this wider resistance landscape. This cancer-specific caution is important because the same CAF label may not carry the same function across organs, treatment histories, or sampling sites. A myCAF-rich area in pancreatic cancer, a stromal invasive front in colorectal cancer, and a post-chemoradiotherapy fibrotic bed in esophageal cancer may all restrict immune access, but they may do so through different mixtures of matrix, chemokine, vascular, and myeloid programs.

Therapy is likely to change CAF states rather than simply act on a static stroma. We use therapy-educated CAFs as shorthand for CAFs reshaped by treatment-induced injury, inflammation, hypoxia, and repair signals. Chemotherapy can trigger tissue injury, inflammatory repair programs, senescence-like phenotypes, extracellular vesicle signaling, and survival-factor release. Radiotherapy can activate TGF-beta and fibrotic repair pathways that remodel ECM over time (19, 20). Immunotherapy imposes selection for spatial and functional immune access, and anti-angiogenic therapy can alter oxygenation, endothelial behavior, and stromal adaptation (21). These pressures need not produce one universal CAF endpoint. A plausible model is convergent function rather than identical phenotype: different therapies and cancer types may induce distinct CAF states that nevertheless produce similar barriers to immune entry, cytotoxic function, and drug penetration. Therefore, CAF evolution should be viewed not only as a histological feature of tumor stroma but also as an adaptive process through which tumors progressively acquire immune-excluded and drug-protected niches.

## Ocular immune privilege and barrier biology: organizing principles for stromal immune restraint

The eye is a classic immune-privileged site where excessive inflammation must be restrained to preserve visual function. Ocular immune privilege depends on coordinated anatomical barriers, blood-retinal and blood-aqueous barrier functions, local immune regulation, restricted immune cell trafficking, and vascular and stromal homeostasis (7–10). These features do not eliminate immunity. Rather, they regulate when, where, and how immune activity can occur in a tissue where inflammatory damage carries high functional cost. The conceptual parallels between ocular immune privilege and CAF-mediated immune exclusion in gastrointestinal cancers are summarized in **Table 1**.

**Table 1:** Conceptual parallels between ocular immune privilege and CAF-mediated immune exclusion in gastrointestinal cancers.

Ocular/barrier concept	Physiological role in the eye	CAF-mediated counterpart in gastrointestinal tumors	Therapeutic consequence
Blood-retinal/blood-aqueous barrier	Limits excessive immune entry	CAF-associated vascular and stromal barrier	Reduced immune infiltration and drug delivery
Local immune restraint	Protects visual tissues from inflammatory damage	TGF-beta-, IL-6-, CXCL12-rich CAF secretome	CD8/NK suppression, Treg/MDSC recruitment
Stromal organization	Maintains tissue structure and optical function	myCAF-driven ECM deposition and stiffness	Physical immune exclusion
Vascular regulation	Controls fluid, oxygen, and immune access	CAF-endothelial crosstalk and abnormal angiogenesis	Impaired trafficking, heterogeneous perfusion
Fibrotic remodeling	Pathologic repair response	Therapy-induced desmoplasia	Persistent resistant niches
Immune-privileged niche	Controlled immune access	Acquired immune-privileged-like tumor niche	ICI resistance and tumor persistence

Ocular pathology also shows how protective barrier and repair programs can become maladaptive. Fibrosis, scarring, inflammatory remodeling, and abnormal angiogenesis illustrate a broader biological principle: barrier architecture plus local immune restraint plus vascular regulation plus stromal remodeling can generate spatially protected niches. This is the principle that is useful for cancer biology.

For CAF-rich tumors, the relevant question is whether treatment-associated stromal programs can co-opt these organizing principles to restrict immune and drug access. The concept is also falsifiable. It would be weakened if CAF-rich post-treatment regions did not show spatial immune exclusion, if immune exclusion occurred independently of stromal barriers, or if CAF reprogramming failed to alter immune access despite changing CAF states.

## CAFs construct acquired immune-privileged-like niches in gastrointestinal tumors

ECM barrier: physical exclusion of immune cells and drugs. myCAF-driven collagen deposition, fibronectin assembly, hyaluronan accumulation, matrix crosslinking, ECM stiffening, and stromal densification can transform tumor stroma into a physical and mechanical barrier (22–24). ECM is not merely a scaffold but an active immune-regulatory barrier. Dense matrix can prevent CD8+ T cells and NK cells from entering tumor nests, increase interstitial pressure, create heterogeneous diffusion paths, and reduce local drug concentrations. The barrier may also change antigen encounter: lymphocytes can remain present in the same biopsy but fail to contact malignant epithelial cells at sufficient frequency or duration. In this setting, residual tumor cells may survive beneath a stromal shield even when systemic therapy reaches the patient.

CAF secretome: biochemical immune restraint. iCAF-like programs can produce TGF-beta, IL-6, CXCL12, VEGF, CCL2, LIF, and extracellular vesicles that shape local immune behavior (3, 4, 13, 14, 25–27). These signals can suppress CD8+ T-cell function, reduce NK-cell activity, recruit Tregs and MDSCs, and polarize macrophages toward immunosuppressive states. In colorectal and pancreatic cancer models, TGF-beta and CXCL12-linked stromal programs have been directly connected with immune exclusion or resistance to PD-1/PD-L1 blockade (3, 4, 25). This biochemical layer matters because immune exclusion is rarely purely mechanical. A permissive matrix without suppressive cytokines may not create durable immune restraint, while a suppressive secretome without spatial structure may diffuse too broadly to define a protected niche. In this sense, the CAF secretome may function as a biochemical counterpart of immune privilege, maintaining local immune restraint within an otherwise non-privileged organ system.

Vascular-barrier remodeling: impaired trafficking and drug delivery. CAFs can alter vessel access through paracrine signals and matrix-generated mechanical stress. In colorectal cancer, CAF-derived WNT2 increased endothelial migration and vessel density, while fibroblast ATF4 supported perivascular CAF activation and angiogenesis in pancreatic and melanoma models (28, 29). In pancreatic cancer, hyaluronan-rich stroma compressed vessels and limited drug delivery. Hyaluronan depletion reopened vessels, whereas angiotensin inhibition reduced collagen and hyaluronan, improved perfusion, and enhanced chemotherapy (30, 31). These results support a coupled stromal-vascular barrier, but the perivascular CAF mechanism still requires cancer-specific validation outside the tested models. Vessel normalization may transiently improve access, yet persistent matrix and CXCL12 gradients can still retain cytotoxic cells at the stromal border (21, 25). Ocular vascular-barrier biology therefore sharpens the question of whether stromal and vascular remodeling jointly determine which immune cells and drugs reach residual tumor.

Spatial immune exclusion: the final phenotype. The acquired immune-privileged-like niche becomes visible when CD8+ T cells are trapped at stromal margins, cytotoxic lymphocytes are excluded from tumor nests, CAF-rich regions colocalize with T-cell exclusion, and Tregs, MDSCs, and tumor-associated macrophages occupy stromal compartments. Spatial structure can also couple fibroblast programs to malignant and myeloid states. Colorectal analyses associated CTHRC1+ CAFs with T-cell exclusion and spatially correlated them with MMP7+ malignant epithelial programs. CellChat further predicted THBS2-SDC4 communication between these populations (32). Single-cell RNA-seq can define cell states, but spatial transcriptomics, multiplex immunofluorescence, imaging mass cytometry, ligand-receptor analysis, and digital pathology are needed to test whether therapy-induced CAF state shifts coincide with increased spatial CD8+ T-cell exclusion (33–36). In clear cell renal cell carcinoma, a cross-tumor analysis spatially colocalized POSTN+ CAF and CCL3+ macrophage programs at invasive margins within hypoxic, fibrotic, CD8+ T-cell-excluded regions (37). Both studies rely mainly on integrated transcriptomic analyses and signaling inference, not perturbation experiments. They therefore support a multicellular niche model but do not establish a causal circuit. Comparable CAF-tumor-myeloid architecture requires validation in gastrointestinal cancers and paired post-treatment tissues. The most informative designs will compare matched pretreatment and post-treatment specimens, because the proposed niche is acquired rather than assumed to be pre-existing. Importantly, the niche should be defined by coordinated function rather than by one marker. A collagen-high region without immune exclusion, a TGF-beta-high region without viable residual tumor, or an abnormal vessel pattern without CAF enrichment would each represent only a partial feature. The full construct requires colocalization of stromal organization, immune restraint, impaired access, and therapy persistence. We define the acquired immune-privileged-like niche as a CAF-organized stromal ecosystem that restricts immune access, suppresses cytotoxic function, and protects tumor cells from therapeutic pressure.

## Therapy-reshaped CAFs as drivers of resistance

Chemotherapy can injure tumor and stromal tissue simultaneously. The resulting repair response may enrich inflammatory or senescence-like CAF states, extracellular vesicle signaling, and paracrine survival factors that support residual tumor cells. In desmoplastic pancreatic cancer, stromal targeting studies showed that the matrix can restrict drug access, while stromal modulation can alter delivery (22, 38, 39). These findings support the idea that chemotherapy resistance can emerge from a combined cancer-cell and stromal-protection program. Pathological complete and major pathological responses after neoadjuvant chemo-immunotherapy were associated with better overall and disease-free survival in resectable ESCC (40). Yet these assessments quantify residual tumor without establishing whether adjacent CAFs, ECM, and immune cells remain an active resistance compartment. For gastrointestinal cancers treated with neoadjuvant chemotherapy or chemoradiotherapy, residual viable tumor should therefore be examined together with the surrounding stroma, not only as isolated malignant cells. In esophageal cancer, prognostic models incorporating nutritional and inflammatory indicators further suggest that systemic inflammatory status and host-tumor interactions may influence postoperative survival and treatment outcomes (41).

Radiotherapy creates a distinct selective pressure. In rectal cancer, clinically fractionated radiation induced DNA damage and IGF1 secretion in CAFs. Irradiated CAFs promoted colorectal cancer-cell survival through IGF1R, and paired specimens showed higher downstream mTOR activity after chemoradiotherapy (42). In lung cancer models, ablative radiation induced persistent senescence and altered the CAF secretome, yet it did not erase CAF-mediated T-cell suppression (43, 44). The direction of these effects is not uniform: the irradiated secretome contained both increased and decreased mediators, while immunosuppression persisted rather than intensified. Radiation also induced TGF-beta-dependent myofibroblast differentiation in primary lung fibroblasts, providing direct support for a fibrotic repair program but not for a gastrointestinal CAF-specific mechanism (45). Thus, direct rectal cancer evidence supports therapy-driven CAF remodeling, whereas evidence for long-lived immune-excluded niches remains a testable inference. Paired pretreatment and post-treatment specimens from rectal, esophageal, gastric, and pancreatic cancers can distinguish an inert scar from an active resistance compartment.

Immunotherapy exposes the importance of spatial access failure. Immune checkpoint inhibitors require T-cell access and cytotoxic competence. CAF-rich stromal barriers may cause resistance even when antigenicity exists, because cytotoxic lymphocytes cannot physically or functionally reach tumor cells (3, 4). Anti-angiogenic therapy adds another layer. Vascular normalization may improve immune entry and drug delivery, but hypoxia and stromal adaptation may also reprogram CAFs (21). CAF-dominant immune-excluded tumors may therefore require combined vascular, immune, and stromal modulation. This combination logic should be driven by phenotype. A matrix-dominant niche may require ECM remodeling, a chemokine-dominant niche may require secretome modulation, and a vessel-dominant niche may require vascular normalization. Therapy does not merely select resistant cancer-cell clones. It also reshapes stromal compartments under therapeutic pressure, enabling CAFs to stabilize immune-excluded and drug-protected niches.

## Translational implications: from conceptual analogy to testable biomarkers

The acquired immune-privileged-like niche should be translated into measurable features. One candidate is a CAF-derived immune-privilege score integrating myCAF ECM genes, iCAF cytokine genes, TGF-beta activity, CXCL12 activity, IL-6 activity, angiogenesis and barrier genes, CD8 exclusion spatial metrics, and Treg, MDSC, and macrophage enrichment. Such a score should not be treated as a universal biomarker at inception. It should be calibrated separately for pancreatic, colorectal, gastric, and esophageal cancers, then tested for prediction of checkpoint blockade resistance, chemoradiotherapy resistance, and benefit from combined anti-angiogenic, immune, and stromal modulation (2–4, 25, 33). Its value would come from connecting cell states to geography. A bulk stromal-high signature may be useful, but a spatial score that distinguishes intratumoral CD8+ penetration from stromal-margin trapping would be more directly aligned with the proposed mechanism.

Validation will require paired pre-treatment and post-treatment samples. The core question is simple: do therapy-induced CAF state shifts coincide with increased spatial CD8+ T-cell exclusion? Single-cell RNA-seq, spatial transcriptomics, multiplex immunofluorescence, imaging mass cytometry, ligand-receptor analysis, digital pathology, and carefully annotated clinical outcomes can answer this question more directly than bulk expression alone (34–36). Multi-scale integration of radiomics, pathomics, spatial transcriptomics, and single-cell data may enable non-invasive assessment of CAF-driven stromal barrier states (46, 47). Recent advances in artificial intelligence for gastrointestinal cancer research further support the integration of multi-omics, imaging, digital pathology, and real-world clinical data for precision patient stratification and biomarker development (48). CAF-rich stroma and ECM remodeling may influence CT or MRI enhancement, stromal density, necrosis distribution, and tumor-margin characteristics, but these associations should be tested cautiously rather than assumed. The most defensible radiomics studies will anchor image features to matched histology and spatial molecular data, then test whether the imaging phenotype predicts therapy response in independent cohorts. Clinically, this argues for tissue collection strategies that preserve geography. Biopsies should record invasive front, stromal margin, tumor nest, necrotic region, and post-treatment scar when feasible, because losing spatial annotation may erase the very phenotype the framework is designed to test.

The therapeutic implication is reprogramming, not eradication. CAFs are heterogeneous, and some stromal programs can restrain tumor progression (49, 50). The goal should not be indiscriminate CAF eradication, but spatially and functionally guided stromal reprogramming. Candidate strategies include modulation of TGF-beta, CXCL12/CXCR4, IL-6/JAK/STAT3, ECM remodeling, vascular normalization, and vitamin D receptor-linked stromal reprogramming, ideally combined with immunotherapy, anti-angiogenic therapy, chemotherapy, or radiotherapy according to spatial phenotype (21, 25, 39). This also changes trial design. Instead of enrolling all stromal-high tumors into one CAF-targeting strategy, future studies could stratify patients by ECM-dominant, secretome-dominant, vascular-barrier-dominant, or mixed acquired immune-privileged-like phenotypes, then test whether the matched stromal intervention improves immune penetration or drug exposure. A minimal validation set would include CAF-state markers, ECM density, vessel features, cytotoxic-cell position, suppressive immune-cell enrichment, and a therapy-response endpoint measured in spatially registered tissue.

## Discussion

Gastrointestinal tumors are not immune-privileged organs, but CAFs may enable them to acquire immune-privileged-like properties under therapeutic pressure. This perspective uses ocular immune privilege as a conceptual model of spatial protection, not as an anatomical equivalence. The strength of the analogy lies in shared organizing principles: barrier architecture, local immune restraint, vascular regulation, stromal remodeling, and controlled trafficking. Its value is greatest when it sharpens experimental design rather than simply renaming immune exclusion. It should remain a working model, not a fixed doctrine, for now.

Several boundaries are important. Current evidence is still indirect for the complete niche concept. Different gastrointestinal cancers may rely on different CAF programs, and findings from pancreatic or colorectal cancer should not be automatically generalized to gastric cancer or esophageal squamous cell carcinoma. Rival explanations, including cancer-cell intrinsic antigen loss, defective antigen presentation, microbiome-driven immune modulation, and systemic immunosuppression, can also generate resistance. The CAF-centered framework is therefore best viewed as a testable hypothesis that can be falsified if therapy-induced CAF state shifts do not align with spatial immune exclusion or therapeutic failure. This caution is not a weakness of the framework. It is the reason to design studies that measure CAF state, vascular access, immune position, and residual tumor survival in the same tissue coordinates.

Future work should ask which therapies most strongly induce immune-privileged-like CAF states, which CAF subsets dominate ECM barrier formation, secretome-mediated immune restraint, and vascular remodeling, and whether spatial CD8+ T-cell exclusion can be reversed by CAF reprogramming. It should also test whether CAF-derived immune-privilege scores predict checkpoint inhibitor resistance and whether radiomics or pathomics can non-invasively capture stromal barrier states. A useful experimental endpoint would be spatial rescue rather than only tumor shrinkage: after stromal intervention, do cytotoxic lymphocytes move from the margin into tumor nests, do suppressive myeloid niches contract, and does drug distribution become more homogeneous? These questions would convert a metaphor into a measurable program. We suggest that future CAF research should move beyond descriptive taxonomy toward spatially guided stromal reprogramming. Reframing CAF-rich gastrointestinal tumors as acquired immune-privileged-like ecosystems may shift the field from descriptive CAF taxonomy toward spatially guided stromal reprogramming strategies.

## Data Availability

The original contributions presented in the study are included in the article/supplementary material. Further inquiries can be directed to the corresponding authors.
